# Supplementary material for: Surveillance of Daughter Micronodule Formation Is a Key Factor for Vaccine Evaluation Using Experimental Infection Models of Tuberculosis in Macaques
Source: Pathogens. 2023 Feb 2;12(2):236. doi: 10.3390/pathogens12020236 (PMC9961649; doi:10.3390/pathogens12020236)
Supplement: Supplementary file 1 [file pathogens-12-00236-s001.zip › pathogens-2085614-Figures and Tables .pdf]

Supplementary File 2 Figures and Tables

Surveillance of Daughter Micronodule Formation is a Key Fac-tor for Vaccine Evaluation Using Experimental Infection Mod-els of Tuberculosis in Macaques

Isabel Nogueira [1,†], Martí Català [2,3,†], Andrew D. White [4], Sally A Sharpe [4], Jordi Bechini [1], Clara Prats [3], Cristina Vi-laplana [5,6,7] and Pere-Joan Cardona [5,6,8,9,*]

[1]Radiology Department, 'Germans Trias i Pujol' University Hospital, 08916 Badalona, Spain

[2]Comparative Medicine and Bioimage Centre of Catalonia (CMCiB), Germans Trias i Pujol Research In-stitute (IGTP), 08916 Badalona, Spain

[3]Escola d'Enginyeria Agroalimentària i de Biosistemes de Barcelona Departament de Física, Universitat Politècnica de Catalunya (UPC)-BarcelonaTech, 08860 Castelldefels, Spain

[4]UK Health Security Agency, Porton Down, Salisbury SP4 0JG, UK

[5]Unitat de Tuberculosi Experimental, Germans Trias i Pujol Research Institute (IGTP), 08916 Badalona, Spain

[6]Centro de Investigación Biomédica en Red de Enfermedades Respiratorias (CIBERES), 28029 Madrid, Spain

[7]Direcció Clínica Territorial de Malalties Infeccioses i Salut Internacional de Gerència Territorial Metropolitana Nord, 08916 Badalona, Spain

[8]Microbiology Department, North Metropolitan Clinical Laboratory, 'Germans Trias i Pujol' University Hospital, 08916 Badalona, Spain

[9]Genetics and Microbiology Department, Universitat Autònoma de Barcelona, 08913 Cerdanyola del Vallès, Spain

*Correspondence: pj.cardona@gmail.com

†These authors contributed equally to this work.

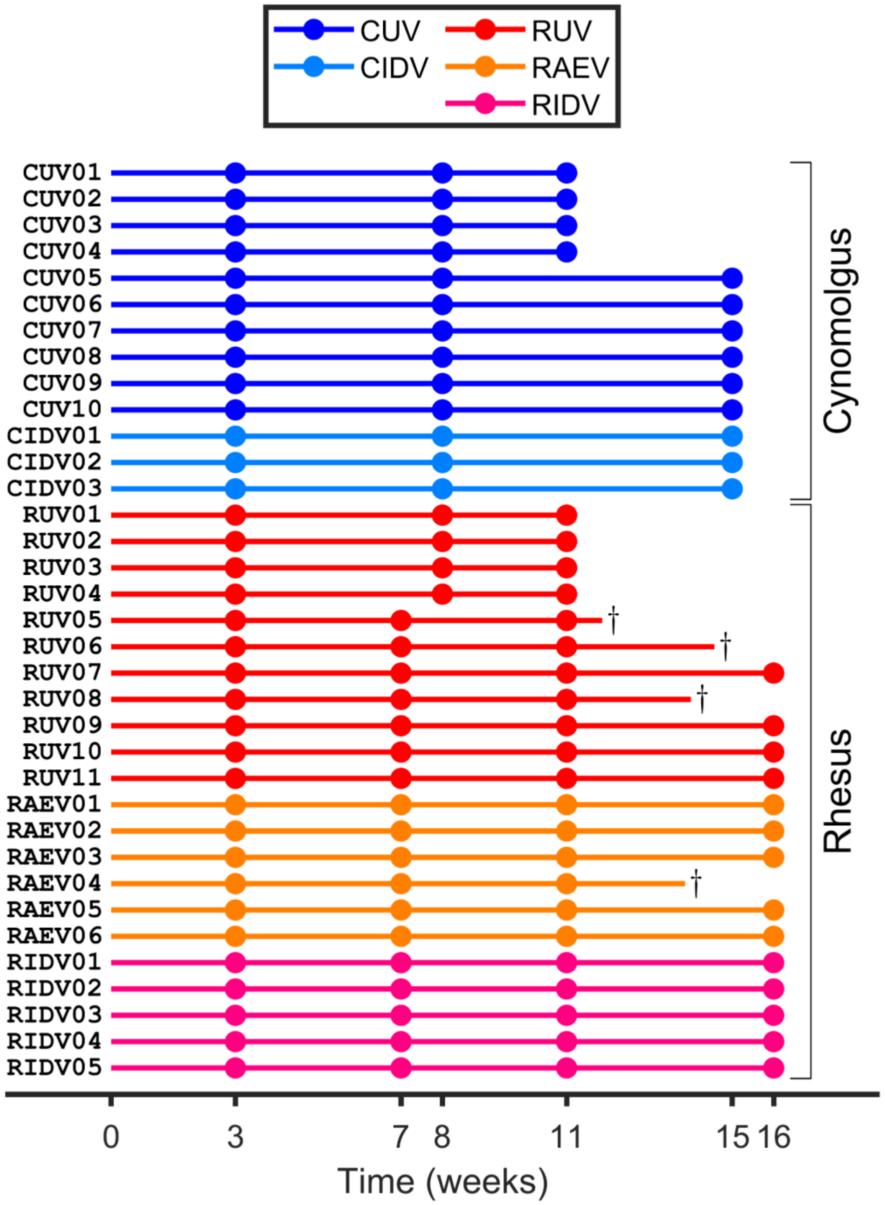

Figure S1. CT scan acquisition for each macaque and experimental groups. A In blue, unvaccinated cynomolgus (CUV). **B** In cyan, intradermal vaccinated cynomolgus (CIDV). **C** In red, unvaccinated rhesus (RUV). **D** In orange, aerosol vaccinated rhesus (RAEV). **E** In purple, intradermal vaccinated rhesus (RIDV). Macaques marked with dagger symbol were euthanized before the planned end of study, according to the welfare monitoring plan.

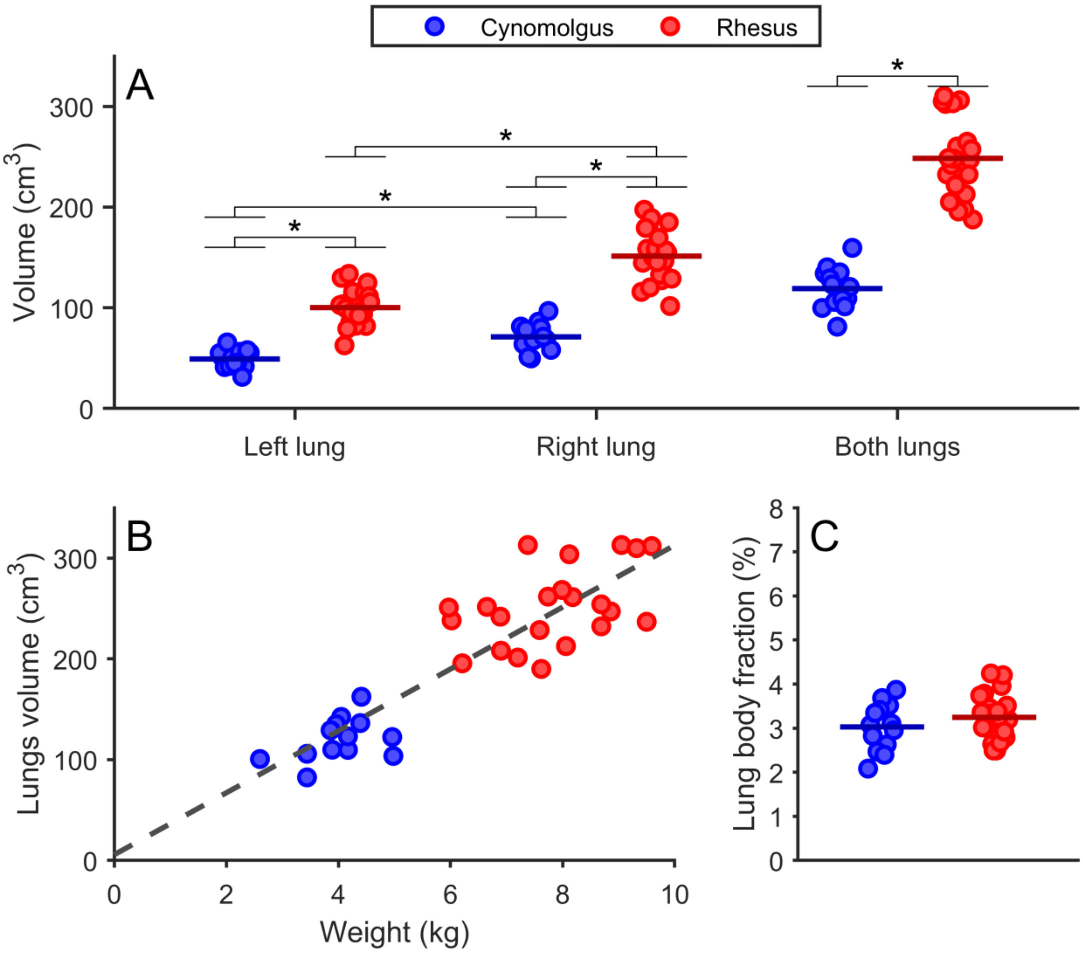

Figure S2. Lung size. Each circle represents a single macaque. Blue circles for cynomolgus and red ones for rhesus. **A** Volume of left, right and both lungs. Horizontal lines mark the mean value for each set of macaques. Significant differences are observed between cynomolgus and Rhesus, also between left lung and right lung in both species. **B** Lungs volume as a function of each macaque weight. Black dotted line is a linear regression for all macaques data: Lungs volume = 30.7 · weight + 5.7. Weight is expressed in kg and lungs volume in cm^3. Goodness of fitting is: $R^2 = 0.79$ **C** Lung body fraction for each macaque. Horizontal lines mark the mean value for each set of macaques. No significant differences are observed between cynomolgus and rhesus.

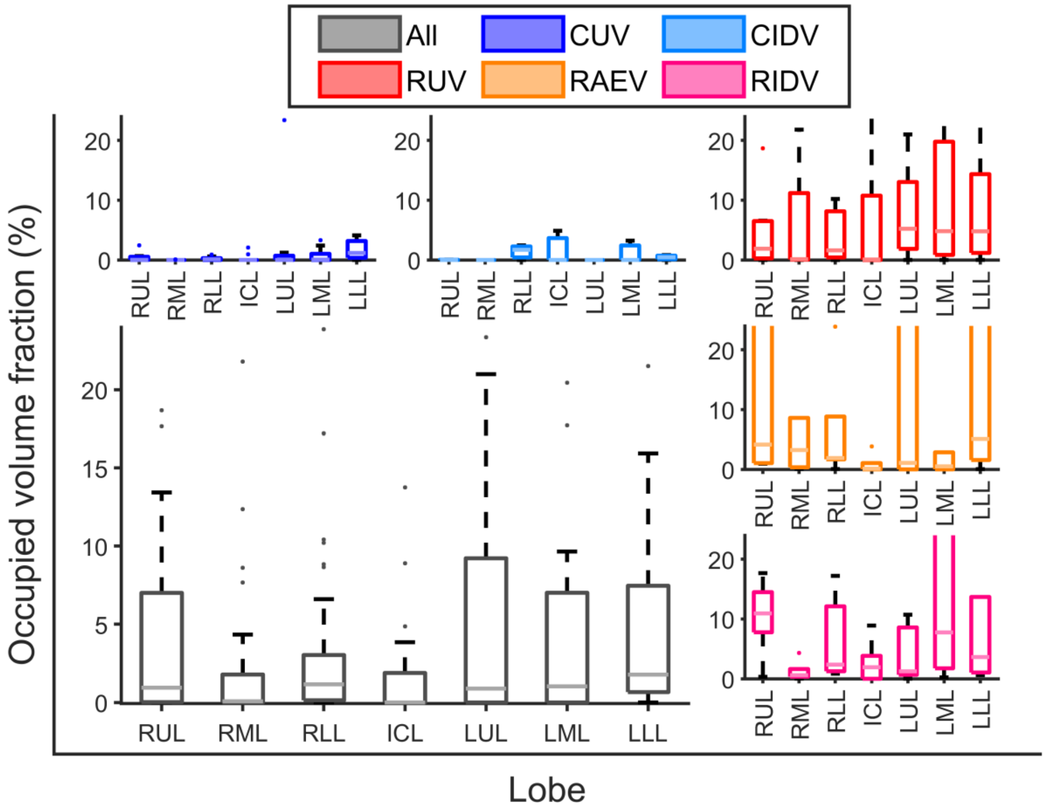

Figure S3. Occupied volume fraction per lobe. Lung lobes: RUL right upper lobe, RM right middle lobe, RLL right lower lobe, IC infracardiac lobe, LUL left upper lobe, LLL left lower lobe. **A** In blue, unvaccinated cynomolgus (CUV). **B** In cyan, intradermal vaccinated cynomolgus (CIDV). **C** In red, unvaccinated rhesus (RUV). **D** In grey, all macaques (all). **E** In orange, aerosol vaccinated rhesus (RAEV). **F** In purple, intradermal vaccinated rhesus (RIDV).

.

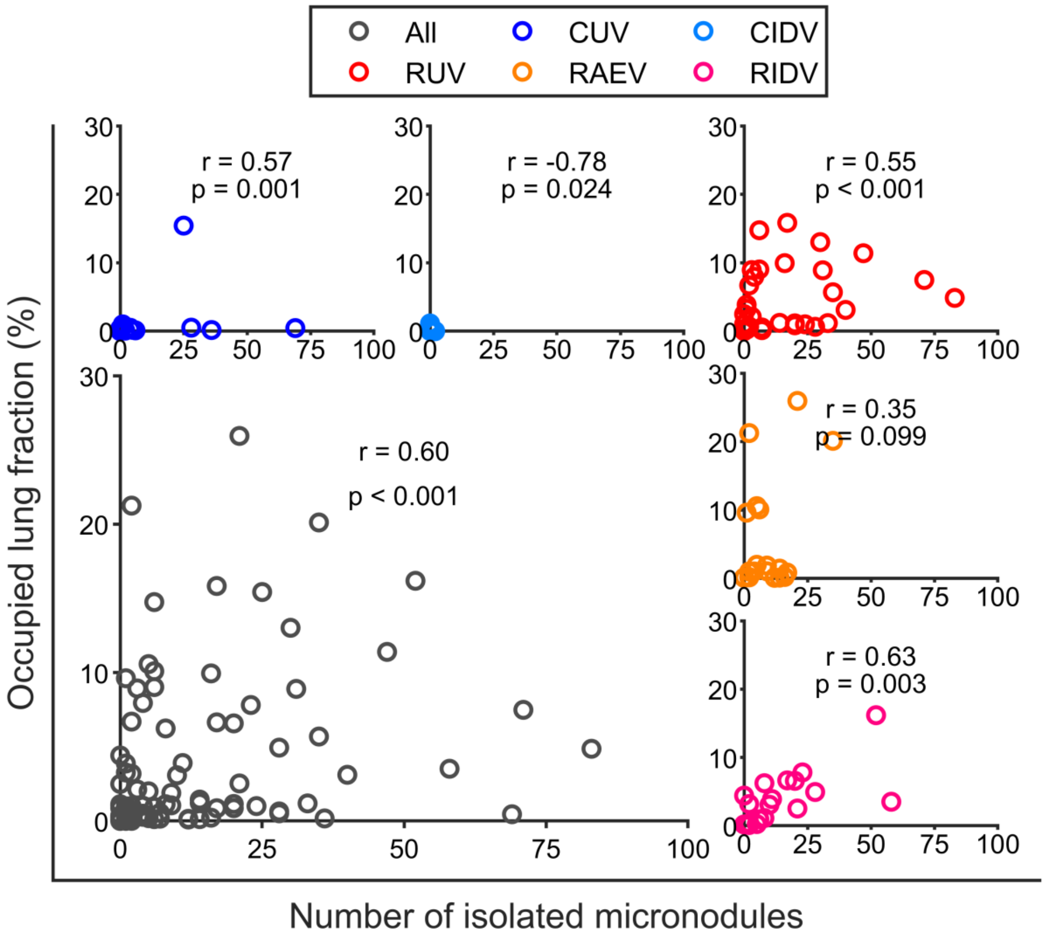

Figure S4. Correlation between the occupied lung fraction and the number of isolated micronodules. Each circle is for a single macaque. Correlation value between both quantities is written as *r.* Significance of correlation value is computed from *p-value* that is shown under correlation value. **A** In blue, unvaccinated cynomolgus (CUV). **B** In cyan, intradermal vaccinated cynomolgus (CIDV). **C** In red, unvaccinated rhesus (RUV). **D** In grey, all macaques (all). **E** In orange, aerosol vaccinated rhesus (RAEV). **F** In purple, intradermal vaccinated rhesus (RIDV).

EG	Consolidations	Transitional lesions	Daughter micronodules	Isolated micronodules
CUV	28	7	75	211
CIDV	10	8	1	3
Cyno.	38	15	76	214
RUV	120	5	428	319
RAEV	79	3	424	137
RIDV	86	2	368	241
Rhesus	285	10	1220	697
All	323	25	1296	911

Table S1. Summary lesions found.

EG: Experimental Group. **CUV**: unvaccinated cynomolgus; **CIDV**: intradermal BCG-vaccinated cynomolgus; **Cynomolgus**: total cynomolgus; **RUV**: unvaccinated rhesus; **RAEV**: aerosol BCG-vaccinated rhesus; **RIDV**: intradermal BCG-vaccinated rhesus; **Rhesus**: total Rhesus; **All**: total macaques

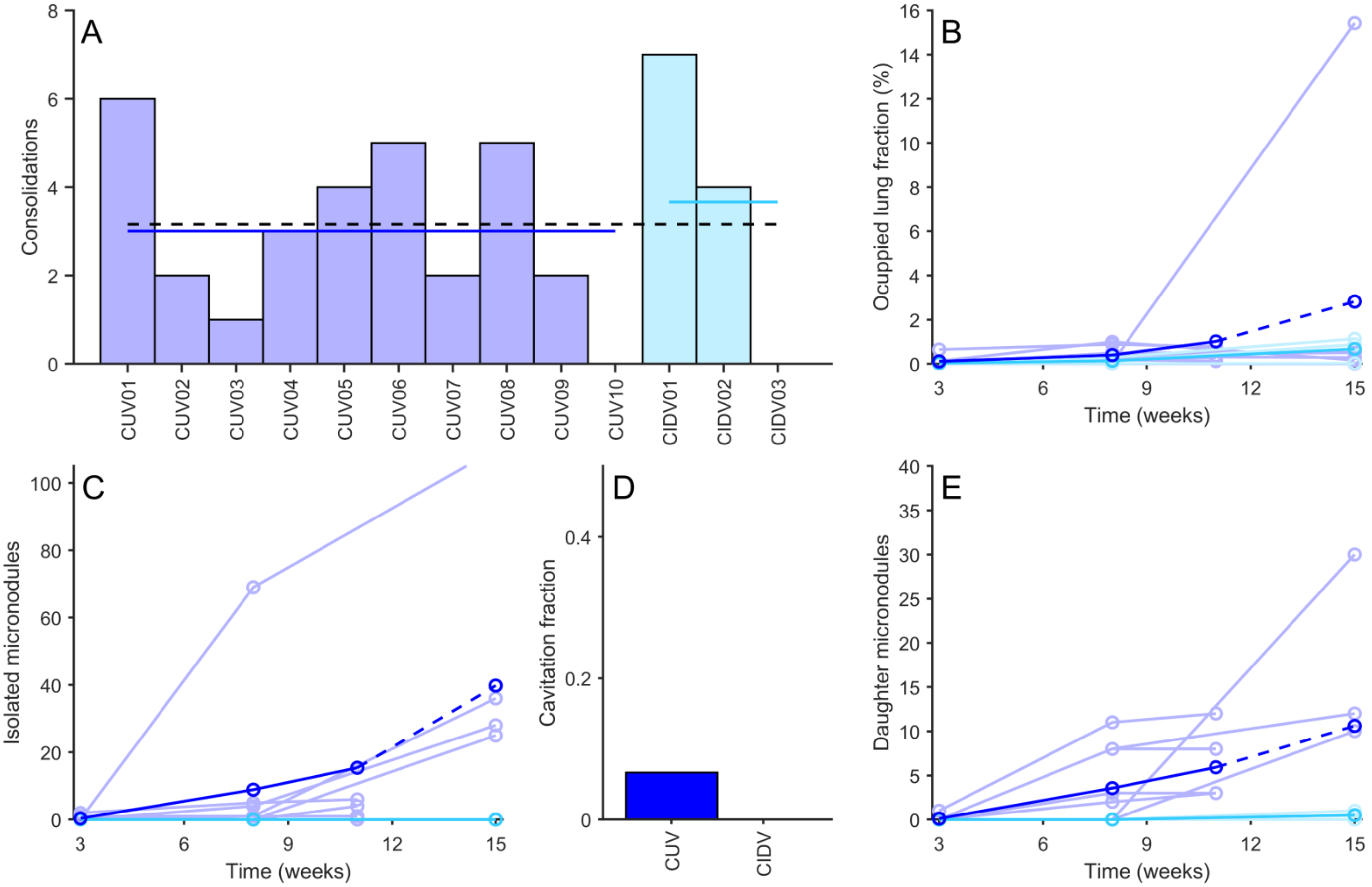

Figure S5. Cynomolgus vaccine effect analysis. **A** Number of identified consolidations for each macaque at the end of the study. In light blue, each unvaccinated cynomolgus (CUV) macaque is represented. In blue, the mean number of consolidations for CUV macaques. In light cyan, each intradermal vaccinated cynomolgus (CIDV) macaque is represented. In cyan, the mean number of consolidations for CIDV macaques. Dotted black line is the mean value for all cynomolgus macaques. No significant differences are observed between the number of consolidations observed in both experimental groups. **B** Occupied lung fraction for each macaque. **C** Number of isolated micronodules for each macaque. **D** Fraction of consolidations that present cavitation in any of the CT scans.

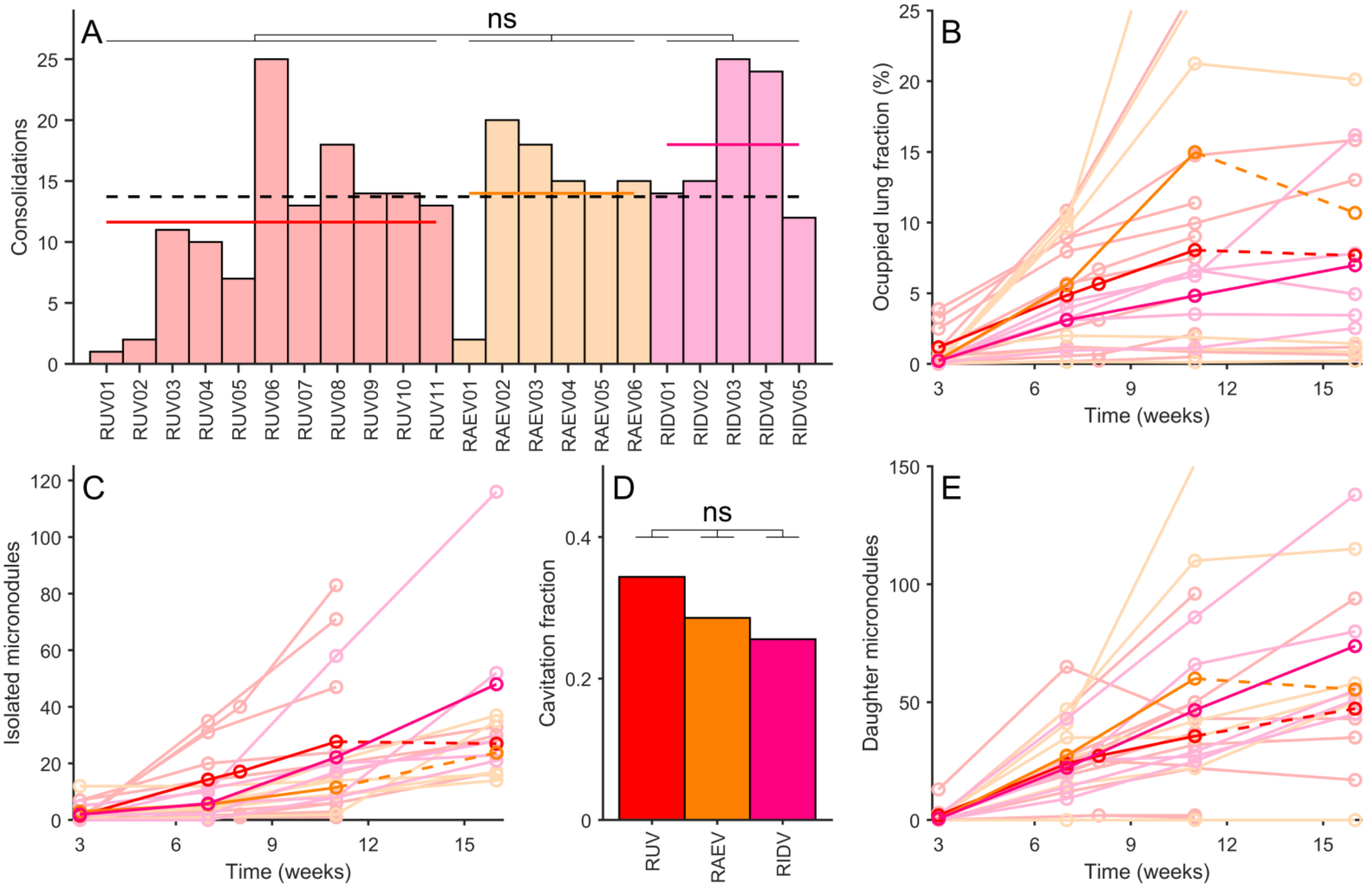

Figure S6. Rhesus vaccine effect analysis. **A** Number of identified consolidations for each macaque at the end of the study. In light red, each unvaccinated rhesus (RUV) macaque is represented. In red, the mean number of consolidations for RUV macaques. In light orange, each aerosol vaccinated rhesus (RADV) macaque is represented. In orange, the mean number of consolidations for RAEV macaques. In light purple, each intradermal vaccinated rhesus (RIDV) macaque is represented. In purple, the mean number of consolidations for RIDV macaques. Dotted black line is the mean value for all rhesus macaques. No significant differences are observed between the number of consolidations observed in both experimental groups. **B** Occupied lung fraction for each macaque. **C** Number of isolated micronodules for each macaque. **D** Fraction of consolidations that present cavitation in any of the CT scans. No significant differences are observed between the cavitation fraction in both experimental groups. **E** Number of daughter micronodules for each macaque. **B, C, E** Each circle represents an experimental measurement from computed tomography (CT) scans. Light lines represent the evolution for each macaque. Darker lines are the mean lines value. Dotted lines are seen when one or more macaques are not considered because they were euthanized or the study ended before that time point.

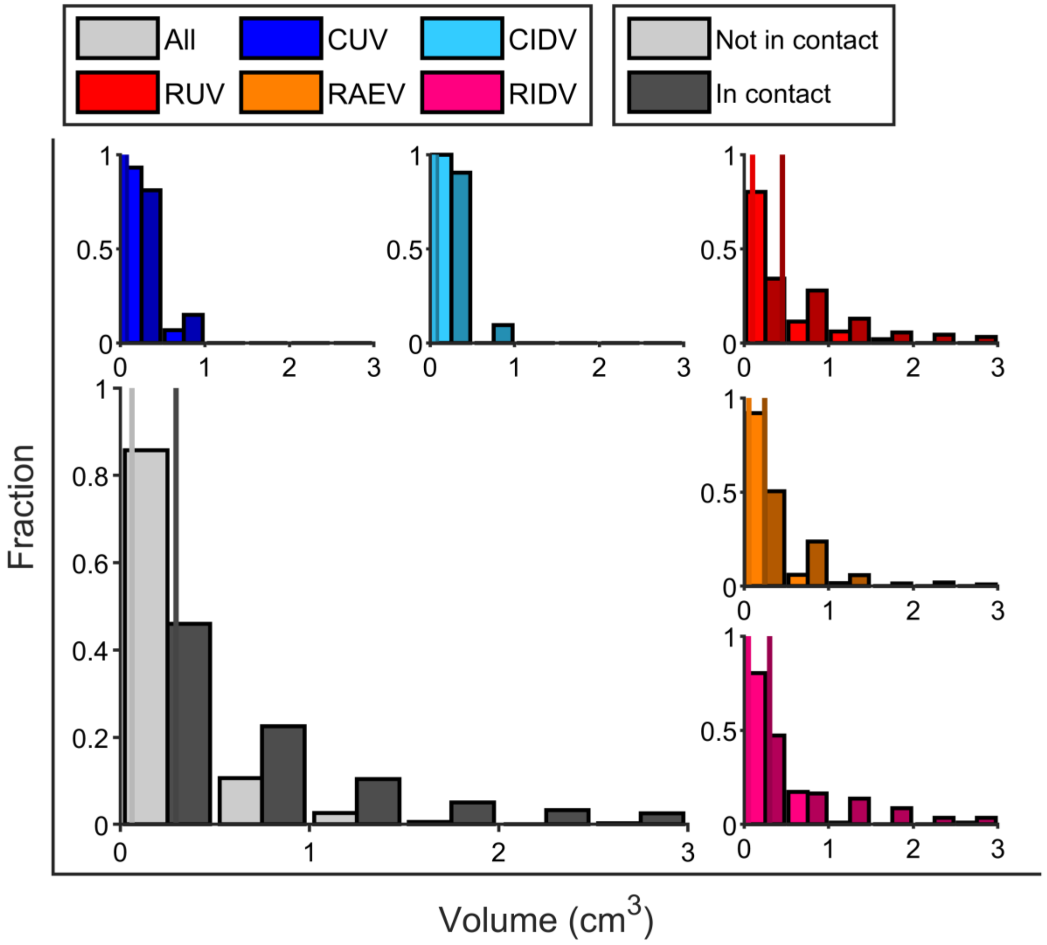

Figure S7. Volume distribution for consolidations in contact (light) and not in contact (dark) with pleura. **A** In blue, unvaccinated cynomolgus (CUV). **B** In cyan, intradermal vaccinated cynomolgus (CIDV). **C** In red, unvaccinated rhesus (RUV). **D** In grey, all macaques (all). **E** In orange, aerosol vaccinated rhesus (RAEV). **F** In purple, intradermal vaccinated rhesus (RIDV).
